# THE ASSOCIATION BETWEEN COGNITION AND METABOLIC BIOMARKERS AMONG PARTICIPANTS FROM THE CHARLS LONGITUDINAL COHORT

**DOI:** 10.1093/geroni/igac059.1589

**Published:** 2022-12-20

**Authors:** Diefei Chen

**Affiliations:** Johns Hopkins Bloomberg School of Medicine, Baltimore, Maryland, United States

## Abstract

Inflammatory or kidney-related metabolic biomarkers have been correlated with cognition and risk for developing dementia among older adults. Using the China Health And Retirement Longitudinal Study, we investigated the cross-sectional correlations between metabolic biomarkers and cognitive outcomes that were ascertained in the same wave, as well as measures lagged by data collection waves. After excluding participants with diagnoses of dementia, we analyzed the association of each biomarker (e.g., White Blood Cell count, Creatinine, Blood Urea Nitrogen, Uric Acid, Cystatin C, C-Reactive protein) with scores from cognitive tests. We then identified biomarkers that showed consistent associations across waves. Having elevated CRP (1-3 mg/L) was positively associated with cognitive test scores; whereas abnormal levels of Creatinine (Low: <0.74 for male and <0.59 for female; High:>1.35 for male and >1.04 for female) were associated with poorer outcomes. These results provide biomarker evidence of the importance of kidney function and inflammation in dementia research.

